# Effects of Renal Denervation on Ouabain-Induced Hypertension in Rats

**DOI:** 10.1155/2024/4763189

**Published:** 2024-06-24

**Authors:** Minna Tang, Jialu Hu, Wenshu Li, Ningzhi Zhang, Sisi Ning, Yan Yan, Zhaoqiang Cui

**Affiliations:** ^1^Department of Cardiology, Zhongshan Hospital, Fudan University, Shanghai Institute of Cardiovascular Diseases, Shanghai, China; ^2^School of Public Health, Fudan University, Shanghai, 200032, China; ^3^Department of Cardiology, Shanghai Changning Tianshan Traditional Chinese Medicine Hospital, Shanghai 200051, China

## Abstract

**Background:**

Ouabain, a Na^+^, K^+^-ATPase inhibitor, is elevated in hypertensive patients. Evidence suggests ouabain contributes to hypertension mainly through activation of the sympathetic nervous system (SNS). Renal nerves play a vital role in the regulation of SNS activity, so we hypothesize that renal denervation may attenuate the development of ouabain-induced hypertension.

**Methods and Results:**

Forty Sprague-Dawley rats were divided into following groups (*n* = 10 each): control group (sham surgery plus intraperitoneal saline injection), RDN group (renal denervation (RDN) plus intraperitoneal saline injection), ouabain group (sham surgery plus intraperitoneal ouabain injection), and ouabain + RDN group (RDN plus intraperitoneal ouabain injection). After eight weeks, compared with the control group, rats in the ouabain group exhibited elevated blood pressure (*P* < 0.05), increased plasma epinephrine, norepinephrine, angiotensin II, and aldosterone levels (*P* < 0.05). These indexes could be significantly ameliorated by RDN. RDN also reduced the thickening of aortic tunica media and downregulated the expression of proliferating cell nuclear antigen (PCNA) in the thoracic aorta induced by ouabain. Masson staining and echocardiography showed that myocardial fibrosis and increased left ventricular mass in the ouabain group could be attenuated by RDN.

**Conclusions:**

The present study reveals that renal nerves play an important role in the development of ouabain-induced hypertension. RDN could inhibit the pressor effect and the myocardial remodeling induced by ouabain potentially via inhibiting catecholamine release and vascular smooth muscle cell proliferation. Clinical studies are needed to explore whether RDN may exhibit better antihypertensive effects on hypertensive patients with high plasma ouabain levels as compared to those with normal plasma ouabain levels.

## 1. Introduction

Renal denervation (RDN), as a novel nonpharmacologic intervention for hypertension [1], is considered to be capable of lowing blood pressure through inhibiting sympathetic nervous system (SNS) and renin-angiotensin-aldosterone system (RAAS) hyperactivity [2, 3]. Clinical studies have demonstrated that RDN could effectively reduce blood pressure in patients with resistant hypertension or uncontrolled hypertension [4–6]. The European Hypertension Society (ESH) issued updated guidelines recently on the role of renal denervation in the hypertension care pathway [7]. The antihypertensive effect of RDN on spontaneous hypertensive rats (SHR) [8] and DOCA-salt hypertensive rats [9] has also been validated. Notably, the Spyral HTN-OFF MED trial, by assessing the efficacy of RDN in the absence of antihypertensive medications to clarify the pure antihypertensive effect of RDN [6], showed that RDN may not achieve equal antihypertensive effects in all hypertensive patients, since the blood pressure did not respond well to RDN in some patients [10]. Therefore, exploring the specific indications for the rational use of RDN therapy in hypertensives is of clinical importance.

Ouabain, a vasopressor hormone of adrenocortical origin, is elevated in hypertensive patients [11, 12]. Approximately 50% of untreated hypertensive patients exhibit high plasma ouabain levels [13], especially those with low or normal plasma renin levels [14]. Hypothalamic and pituitary ouabain content was also elevated in Dahl salt-sensitive hypertensive rats [15]. As a Na^+^, K^+^-ATPase inhibitor, ouabain contributes to elevated blood pressure mainly through activation of the sympathetic nervous system [16]. Intracerebroventricular injection of antibody Fab fragments against ouabain could effectively inhibit excess sympathetic activation and prevent the elevation of blood pressure in Dahl salt-sensitive hypertensive rats [17]. Hypertensive animal models can be established through intraperitoneal injection of ouabain [18–22].

Additionally, ouabain could also induce cardiac remodeling in rats independent of blood pressure [21]. The proliferation of collagen fibers among myocardial cells, as well as mitochondrial proliferation and swelling in myocardial cells, was observed under electron microscopy in ouabain-treated rats [22] without obviously elevated blood pressure [21]. Mechanisms involved in ouabain-induced cardiac remodeling have not been completely elucidated.

Evidence indicated that ouabain has a complicated interaction with other neurohormonal systems [16, 17], leading to SNS and RAAS activation, which may contribute to elevated blood pressure as well as cardiac dysfunction and structural damage [23]. Previous studies suggested that RDN could inhibit SNS and RAAS activation [2, 3]. However, the efficacy of RDN on ouabain-induced hypertension remains elusive. Hence, we speculate that inhibition of SNS and RAAS overactivity through RDN may exert beneficial effects on ouabain-induced hypertension and cardiac remodeling. The present study thus evaluated the effects of bilateral RDN on ouabain-induced hypertension in rats.

## 2. Materials and Methods

### 2.1. Animals

Male Sprague-Dawley rats, weighing 160–190 g, were purchased from the Experimental Animal Center of Zhongshan Hospital Fudan University (Shanghai, China). Rats were housed in cages with controlled temperature and humidity, and under a 12/12 h light/dark cycle, and they were allowed free access to food plus water. Forty Sprague-Dawley rats were randomly divided into four groups (*n* = 10 each): the control group (sham surgery plus intraperitoneal saline injection), the RDN group (RDN plus intraperitoneal saline injection), the ouabain group (sham surgery plus intraperitoneal ouabain injection of 27.8 *μ*g/kg/d), and the ouabain + RDN group (RDN plus intraperitoneal ouabain injection of 27.8 *μ*g/kg/d). Intraperitoneal injection of saline or ouabain was daily administrated for eight weeks after the surgery. Blood pressure was measured at baseline, four weeks, and eight weeks after injection. After eight weeks of saline or ouabain injection, echocardiography was performed. Finally, rats were euthanized, and blood samples, renal arteries, thoracic aorta, heart tissue, and renal cortex were collected for various analyses. All animal studies were conducted according to the National Institutes of Health (NIH) Guide for the Care and Use of Laboratory Animals, and the experimental procedures were approved by the Animal Ethics Committee at the Zhongshan Hospital, Fudan University.

### 2.2. Renal Denervation

Total denervation of the kidneys was performed as previously described [24, 25]. Rats were anesthetized using pentobarbital sodium (50 mg/kg intraperitoneally). Bilateral renal arteries were exposed through a midline abdominal incision. All visible renal nerves around the renal artery were cut. Then, the renal artery and vein were repeatedly wiped by a cotton swab soaked in 10% phenol in ethanol to fully remove the renal nerves. Sham surgery was performed by exposing bilateral renal nerves without physical disruption of the area.

### 2.3. Echocardiography

Doppler echocardiography (Vevo 2100, VisualSonics Inc., Toronto, Canada) was performed to assess cardiac function in rats (*n* = 10 each) under anesthesia (inhalation of 3.0% isoflurane and oxygen at a rate of 1 L/min). A long-axis view of the left ventricle (LV) was obtained at the level of the mid-papillary level. Three sequential cardiac cycles of two-dimensional images were recorded. Interventricular septal thickness at end-systole (IVSs), interventricular septal thickness at end-diastole (IVSd), posterior wall systolic thickness of LV (LVPWs), posterior wall diastolic thickness of LV (LVPWd), LV end-systolic diameter (LVESd), and LV end-diastolic diameter (LVEDd) were measured. LV ejection fraction (LVEF), LV fractional shortening (LVFS), and LV mass were calculated. The calculation formula for LV mass is 0.8 *∗* 1.04 *∗* [(LVEDd + IVSd + LVPWd)^3^ − (LVEDd)^3^] + 0.6.

### 2.4. Blood Pressure Measurement

Systolic (SBP) and diastolic blood pressures (DBP) were measured using the tail-cuff method based on volume pressure recording (VPR) technology (CODA 8; Kent Scientific Corporation, Torrington, CT). In brief, rats (*n* = 10 each) were fixed in a tube-shaped holder on the Kent warming panel for 10–20 minutes/day for 3–5 consecutive days to make them acclimate to the measurement. After this training, rats usually remained relatively unperturbed in the holder on the day of testing. We ensured that the procedure was conducted in a quiet environment. After 5–10 min of stabilization in the chamber, blood pressure measuring was repeated 10 times upon the temperature at the base of the tail reaching 32°C. The normal measuring process was screened out by blood measuring software system. The mean blood pressure of repeated measurements was reported.

### 2.5. Histological Examinations

Renal artery and heart tissue paraffin sections were prepared by routine procedures [26]. In brief, renal artery and heart samples of rats (*n* = 10 each) were isolated and fixed in 4% paraformaldehyde and then embedded in paraffin and sectioned at a thickness of 4 *μ*m. Renal artery sections were stained with hematoxylin-eosin (HE; G1005, Servicebio, Wuhan, China) and tyrosine hydroxylase (TH; ab137869, Abcam, MA, USA) according to standard protocol. Thoracic aorta sections were stained with *α*SMA (GB111364, Servicebio, Wuhan, China) and Ki-67 (GB151142, Servicebio, Wuhan, China). Heart tissue sections were stained with Masson staining (G1006, Servicebio, Wuhan, China). The percentage of fibrotic area accounting for the total myocardial area and the thickness of aortic tunica media was measured in five random different fields per section using Image J. The ratio of Ki-67 positive cells to total number of cells in aortic tunica media was calculated in five random fields per section by an investigator blinded to the study. Micrographs were captured using a Leica fluorescence microscope (DM2500).

### 2.6. Plasma and Renal Cortex Hormones Measurement

At the end of the experiment, blood samples of rats (*n* = 10 each) were collected from the inferior vena cava. We used commercial assay kits to measure plasma renin activity (PRA), angiotensin II, aldosterone, norepinephrine, and epinephrine levels as well as renal cortex norepinephrine concentration. Plasma renin activity, angiotensin II, and aldosterone (KIP1531, KIPERB320, RVR-CW-100, Beijing North Institute of Biotechnology Corporation, Beijing, China) were measured by radioimmunoassay (RIA). Plasma norepinephrine and epinephrine and renal cortex norepinephrine (Zhejiang Designs Diagnostics Technology Corporation, Zhejiang, China) were measured by liquid chromatography-tandem mass spectrometry (LC-MS/MS) [27]. In brief, plasma from blood samples (containing EDTA) was collected. Solid-phase extraction was conducted for pretreatment of plasma samples. LC-MS/MS was performed using the SCIEX Triple Quad™ 4500MD system, and data were automatically processed by the application manager.

### 2.7. Western Blotting Analysis

At the end of the experiment, thoracic aortas of rats (*n* = 10 each) were collected. Total protein was extracted from the thoracic aorta, and the protein concentration was determined using the BCA method (P0012, Beyotime Institute of Biotechnology, Shanghai, China). Western blotting was performed as previously described [28]. Antibodies used for this experiment were as follows: proliferating cell nuclear antigen (PCNA; 13110, 1 : 1,000, Cell Signaling Technology Inc., MA, USA); *β*-tubulin (2146, 1 : 1,000, Cell Signaling Technology Inc., MA, USA).

### 2.8. Statistical Analyses

Data with a normal distribution were exhibited as mean ± standard error of the mean (SEM). SPSS (version 26) was used for the statistical analysis of the data. Comparison between groups was analyzed using one-way ANOVA followed by the Tukey post hoc test. Repeated measurements between groups were analyzed by two-way repeated measures ANOVA followed by the Tukey post hoc test. *P* < 0.05 was considered statistically significant.

## 3. Results

**Figure 1:**
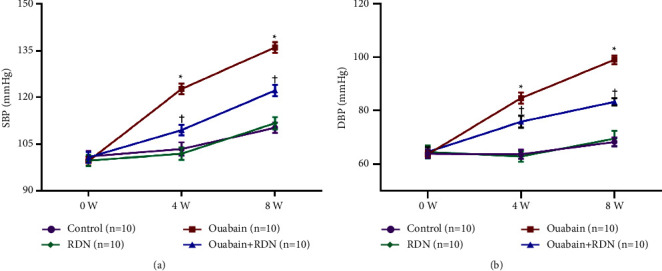
Caudal arterial pressure of rats. (a) Systolic blood pressure of rats at baseline, four weeks, and eight weeks after ouabain or saline injection. (b) Diastolic blood pressure of rats at baseline, four weeks, and eight weeks after ouabain or saline injection. Two-way repeated measures ANOVA, *n* = 10/group. Data presented as mean ± SEM; ^*∗*^*P* < 0.05 versus the control group; ^†^*P* < 0.05 versus the ouabain group. SBP, systolic blood pressure; DBP, diastolic blood pressure.

As expected, bilateral total RDN effectively reduced ouabain-induced hypertension (Figure 1).

### 3.1. Verification of Renal Denervation

**Figure 2:**
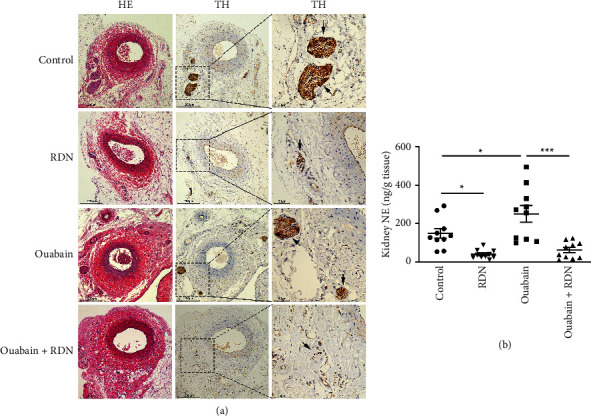
Verification of renal denervation. (a) HE and TH staining of renal nerves. (b) Quantification of NE concentration per gram of renal cortex of rats from different groups. One-way ANOVA, *n* = 10/group. Data presented as mean ± SEM; ^*∗*^*P* < 0.05; ^*∗∗*^*P* < 0.01; ^*∗∗∗*^*P* < 0.001. RDN, renal denervation; HE, hematoxylin-eosin staining; TH, tyrosine hydroxylase; NE, norepinephrine.

Denervation of the renal nerve was verified by HE staining and TH immunohistochemical staining of renal artery sections as well as by the kidney norepinephrine measurement. TH immunohistochemical staining exhibited significantly less positive staining inside the renal nerve in the ouabain + RDN group than in the ouabain group (Figure 2(a)). Kidney norepinephrine content in the ouabain group was significantly higher than that in the control group (*P* < 0.05). Kidney norepinephrine decreased by approximately 60% in the ouabain + RDN group compared with the ouabain group (*P* < 0.05) (Figure 2(b)).

### 3.2. RDN Inhibited Ouabain-Induced Release of Catecholamine

**Figure 3:**
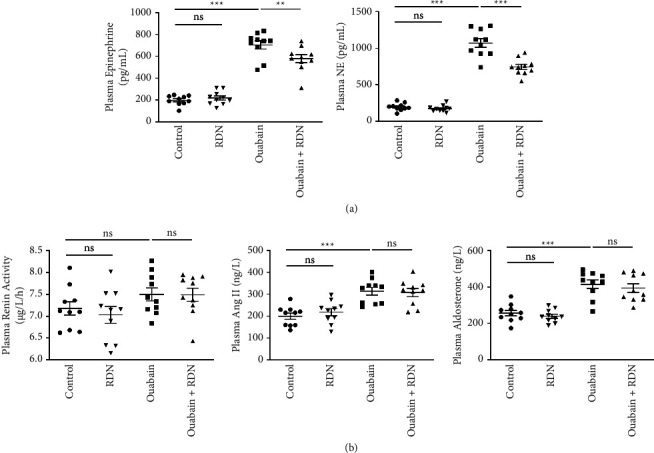
Plasma hormone levels of rats after administration of ouabain for eight weeks. (a) Quantification of plasma epinephrine and norepinephrine levels of rats after administration of ouabain for eight weeks. (b) Quantification of plasma renin, angiotensin II, and aldosterone levels of rats after administration of ouabain for eight weeks. One-way ANOVA, *n* = 10/group. Data presented as mean ± SEM; ^*∗*^*P* < 0.05; ^*∗∗*^*P* < 0.01; ^*∗∗∗*^*P* < 0.001. Ang II, angiotensin II; NE, norepinephrine.

To explore the effect of RDN on the SNS and RAAS in rats with ouabain-induced hypertension, plasma epinephrine, norepinephrine, renin, angiotensin II, and aldosterone were tested after the administration of saline or ouabain for eight weeks. The plasma epinephrine levels were 197.47 ± 14.09 pg/mL, 218.11 ± 19.17 pg/mL, 704.79 ± 37.34 pg/mL, and 577.92 ± 36.84 pg/mL, and the plasma norepinephrine levels were 195.75 ± 16.05 pg/mL, 172.08 ± 13.88 pg/mL, 1070.73 ± 58.67 pg/mL, and 743.96 ± 35.67 pg/mL in the control, RDN, ouabain, and ouabain + RDN groups, respectively. The plasma epinephrine and norepinephrine levels increased significantly in the ouabain group compared with the control group (*P* < 0.05), while rats from the ouabain + RDN group had lower plasma epinephrine and norepinephrine levels than those from the ouabain group (*P* < 0.05) (Figure 3(a)). No significant difference in plasma renin activity was found among the four groups. Rats from the ouabain group and ouabain + RDN group had higher plasma angiotensin II and aldosterone levels than those from the control group (*P* < 0.05), but there were no significant differences in plasma angiotensin II and aldosterone levels between the ouabain and ouabain + RDN groups (*P* > 0.05) (Figure 3(b)).

### 3.3. RDN Inhibits Ouabain-Induced Vascular Smooth Muscle Cell Proliferation

**Figure 4:**
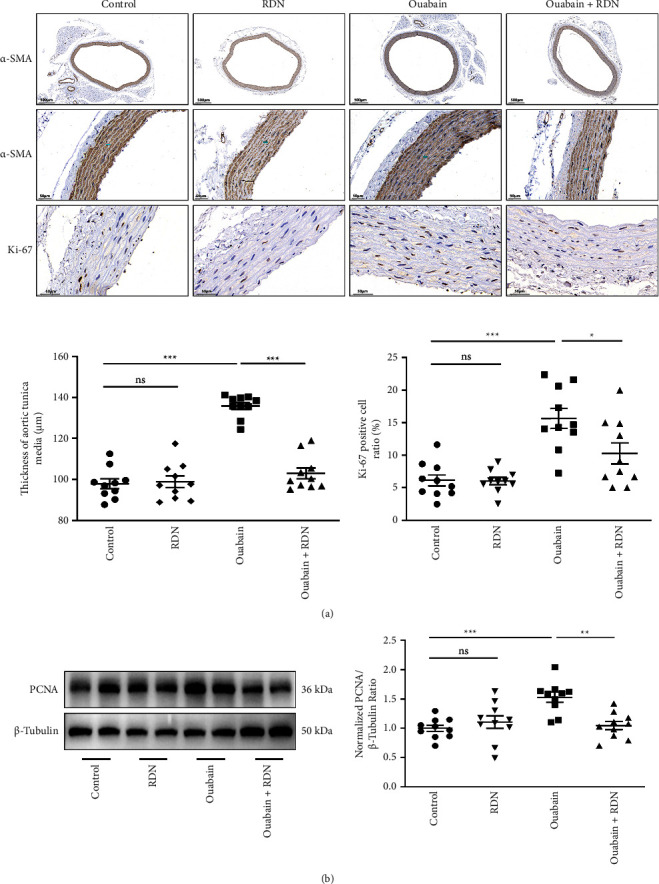
(a) *α*SMA and Ki-67 staining of the thoracic aorta. (b) Representative images and relative protein expression of PCNA in thoracic aorta of rats from different groups. One-way ANOVA, *n* = 10/group. Data presented as mean ± SEM; ^*∗*^*P* < 0.05; ^*∗∗*^*P* < 0.01; ^*∗∗∗*^*P* < 0.001. PCNA, proliferating cell nuclear antigen.

To clarify the underlying mechanism by which RDN lowers blood pressure, we examined the role of RDN in the proliferation of vascular smooth muscle cells. The thickness of aortic tunica media was measured, the Ki-67 positive cell ratio in aortic tunica media was calculated, and the protein expression levels of PCNA in the thoracic aorta of rats were determined (Figure 4). The results showed that the thickness of aortic tunica media, the Ki-67 positive cell ratio in aortic tunica media, and the expression levels of PCNA were significantly increased in the ouabain group compared with the control group (*P* < 0.05) but were significantly decreased in the ouabain + RDN group compared with the ouabain group (*P* < 0.05).

### 3.4. RDN Mitigates Cardiac Remodeling Induced by Ouabain

**Figure 5:**
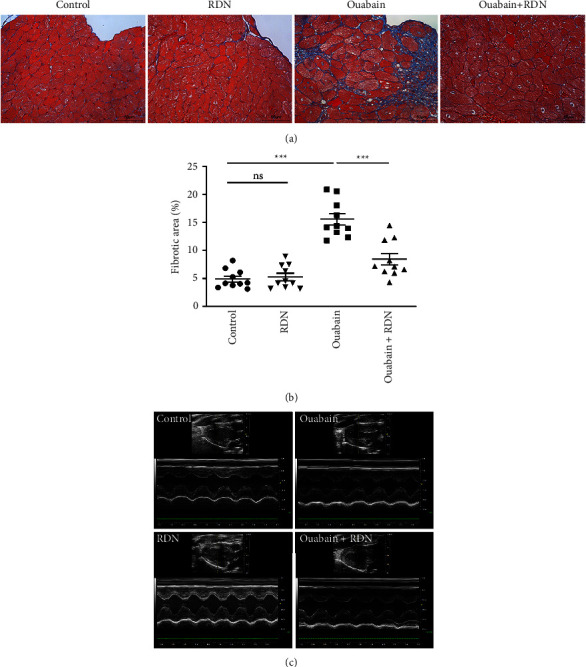
Renal denervation attenuated cardiac remodeling induced by ouabain. (a) Representative photomicrographs of Masson staining. (b) Quantitative analysis of Fibrotic area (blue area in Masson staining). One-way ANOVA, *n* = 10/group. (c) Representative echocardiography images of rats in different groups. Data presented as mean ± SEM. ^*∗*^*P* < 0.05; ^*∗∗*^*P* < 0.01; ^*∗∗∗*^*P* < 0.001.

The cardiac function of rats was assessed by Doppler echocardiography eight weeks after the beginning of ouabain or normal saline administration. Compared with the control group, the ouabain group had significantly increased LV mass (*P* < 0.05). LV mass in the ouabain + RDN group was also greater than that in the control group but smaller than that in the ouabain group; however, the differences were not statistically significant (*P* > 0.05). There were no significant differences in IVSD, IVSS, LVEF (%), LVEF (%), LVEDd, LVEDs, LVPWd, or LVPWs among the four groups (Table 1). Cardiac Masson staining showed that rats from the ouabain group displayed apparent myocardial fibrosis (Figure 5(a)). However, the cardiac fibrotic area in cardiac tissue decreased significantly in the ouabain + RDN group compared with the ouabain group (*P* < 0.05) (Figure 5(b)).

## 4. Discussion

In this study, we successfully established hypertensive rat models through intraperitoneal injection of ouabain. According to the previous pharmacokinetic results for ouabain in rats, the dosage of ouabain administered in this study was determined as 27.8 *μ*g/kg/d [18]. The doses infused were estimated to increase the plasma ouabain concentration by 0.5–1.0 nmol/l above the normal physiological level (approximately 0.3–0.9 nmol/L) [29], thus leading to an ouabain concentration similar to that in pathological conditions such as hypertension and heart failure (approximately 0.9–1.8 nmol/L) [30]. Intraperitoneal injection of ouabain daily for consecutive eight weeks has been proven to effectively increase blood pressure in SD rats [21, 22].

The established hypertensive rat model exhibited elevated plasma epinephrine, norepinephrine, angiotensin II, and aldosterone but normal renin levels, suggesting the activation of the SNS and RAAS, which was consistent with previous studies [31–36]. It was previously found that intravenous injection of sympathetic ganglion blocker hexamethyl bromide ammonium normalized blood pressure in rats treated with ouabain [31], which demonstrated that SNS activation played a key role in the development of ouabain-induced hypertension. In addition, the study indicates that ouabain could also augment the transmission of preganglionic sympathetic nerve signals to postganglionic fibers and increase the sensitivity of blood vessels to sympathetic excitation by enhancing the duration of long-term potentiation (LTP) in sympathetic neurons [32] as well as increase norepinephrine overflow from the sympathetic nerve endings [33, 34]. Meanwhile, evidence suggested that ouabain might activate the central sympathetic nervous system. Peripheral infusion of ouabain increased the secretion of aldosterone [37] which further stimulated ouabain secretion from hypothalamus [38], thus augmenting central sympathetic outflow. Additionally, peripheral administration of ouabain could also increase Ang II content in hypothalamus [39] and activate afferent renal nerve [40], consequently enhancing central sympathetic nervous activity [41, 42]. In our study, ouabain-induced hypertensive rats exhibited elevated Ang II and aldosterone levels but normal plasma renin activity. Studies showed that ouabain could increase Ang II secretion from human kidney proximal tubule cells and adrenal cells [43], as well as stimulate the growth and steroidogenic capacity of adrenal zona glomerulosa [37], thus resulting in elevated plasma Ang II and aldosterone levels. Increased aldosterone afterward conversely inhibits renin secretion through negative feedback mechanism, leading to reduced renin activity. Likewise, previous studies found that ouabain and aldosterone are coelevated in the circulation in patients with essential hypertension [35] and the plasma aldosterone levels increased markedly in rats treated with ouabain [36], suggesting RAAS activation induced by ouabain.

Cardiac hypertrophy and myocardial fibrosis are evidenced in ouabain-induced hypertensive rats. Our results are in general agreement with previous studies. It has been proved that ouabain promoted cardiomyocyte hypertrophy in vitro [44], and a high ouabain level correlated with elevated left ventricular mass in patients with hypertension [13]. There are two possible explanations for these pathological changes induced by ouabain. First, elevated blood pressure leads to increased heart afterload thus promoting cardiac remodeling in rats. In addition, ouabain, as a sodium-potassium pump inhibitor, can enhance myocardial contraction by regulating intracellular calcium signaling in cardiomyocytes [45]. Therefore, enhanced myocardial contraction and pressure overload may both result in myocardial remodeling in ouabain-induced hypertensive rats.

Our study showed that preventive RDN, removing both afferent and efferent renal nerve fibers, could reduce blood pressure by about 20 mmHg. This result indicated that the renal nerve plays an important role in the development of ouabain-induced hypertension. It is known that renal nerves are critical to the regulation of blood pressure. Stimulation of the afferent renal nerve activates the paraventricular nucleus of hypothalamic neurons and elicits increased sympathetic nerve activity [46] while activation of the efferent renal nerve increases sodium reabsorption in the renal tubule and stimulates renin secretion with subsequent RAAS activation. These pathophysiological processes mediated by renal nerves further influence blood pressure. Our results demonstrated preventive RDN reduced the high plasma catecholamine levels in rats treated with ouabain. One possible implication of this is that RDN restrained the activation of renal nerves induced by ouabain, thus inhibiting SNS activation and thereby preventing the rise of blood pressure. Kopp et al. found that renal pelvic perfusion with ouabain increased afferent renal nerve activity [40] which also supports our results. However, our results did not support the notion from other studies that RDN could inhibit the activation of RAAS(2) since RDN did not reduce the elevated plasma angiotensin II and aldosterone levels in rats treated with ouabain in our study. Studies suggested that ouabain increased the secretion of Ang II in cultured human kidney proximal tubule cells and adrenal cells [43] and selectively stimulated the growth and steroidogenic capacity of adrenal zona glomerulosa [37], thus resulting in elevated plasma Ang II and aldosterone levels, which, however, could not be attenuated by RDN. Overall, RDN did not reduce the elevated plasma angiotensin II and aldosterone levels but inhibited catecholamine secretion in rats treated with ouabain, indicating RDN mainly reduced sympathetic outflow rather than influence RAAS in this hypertensive model. In light of this, the antihypertensive effect of RDN on ouabain-induced hypertensive rats may be mainly due to ablation of afferent renal nerves. This hypothesis needs to be further verified by conducting selective afferent renal nerve ablation on this model. In addition, we found that RDN inhibited the proliferation of vascular smooth muscle cells in the thoracic aorta of rats treated with ouabain, indicating excessive vascular remodeling and enhanced vasoconstriction potentially exist in ouabain-induced hypertensive rats, whereas RDN could attenuate those pathological processes. The reduced vascular smooth muscle cell proliferation might be attributed to the decreased release of neuropeptide *Y* and norepinephrine by the peripheral sympathetic nerve. These two hormones were proven to stimulate vascular smooth muscle cell proliferation [47, 48] and were decreased in heart tissue after RDN [49]. However, the change in these two hormones' contents in vascular tissue is unknown and needs to be further determined.

Notably, RDN could not completely inhibit the pathways mediating the hypertensive effect of ouabain since the blood pressure of rats from the ouabain + RDN group still increased slightly in our study, implying there are other pathways that contribute to the pressor effect of ouabain which cannot be blocked by RDN, as it has been reported that ouabain could also directly augment vasoconstriction through regulating intracellular calcium signaling [45]. By inhibiting sodium-potassium pumps, ouabain increases intracellular Na^+^, resulting in decreased activity of the sarcolemmal Na^+^/Ca^2+^ exchanger and reduced Ca^2+^ extrusion. As a result, intracellular Ca^2+^ accumulates and is taken up by the sarcoplasmic reticulum, which, upon activation, releases more calcium and eventually increases the vascular smooth muscle tone [49]. It is probably a reason why RDN incompletely inhibited ouabain-induced hypertension in our study.

In this study, preventive RDN also attenuated myocardial fibrosis induced by ouabain. The protective effect of RDN on myocardial remodeling may be partly attributed to blood pressure reduction. Besides, our study suggested that RDN reduced the elevated circulating catecholamine levels induced by ouabain thus might mitigate the myocardial fibrosis caused by sympathetic hyperactivity.

While writing our manuscript, Lai et al. published their results regarding the effects of renal denervation on endogenous ouabain in spontaneously hypertensive rats [50]. The similarities and differences between the two studies are as follows: the similarity between Lai's paper and this paper is that we both proved that RDN exerted an antihypertensive effect on hypertension associated with ouabain. The major difference between the two studies is the hypertensive rat model: spontaneously hypertensive rats versus ouabain-induced hypertensive rats. Lai et al. explored the influence of RDN on ouabain secretion in spontaneously hypertensive rats, and they found RDN could inhibit ouabain secretion in this rat model thus reducing blood pressure [50]. However, we have explored the effect of RDN on ouabain-induced hypertensive rats. In our study, the antihypertensive effect of RDN was confirmed without changing the ouabain content in this hypertensive rat. The two studies indicated that RDN not only inhibited ouabain secretion but also disrupted the ouabain-mediated pressor effect.

Although there are important discoveries revealed by this study, there are also limitations. First, the blood pressure of this ouabain-induced hypertensive rat model only elevated moderately. Second, we evaluated sympathetic tone by measuring plasma catecholamine content rather than testing sympathetic nerve activity. Moreover, previous studies indicated ouabain is mainly acting central nervous system to drive the SNS activity leading to hypertension. Therefore, a hypertensive rat model built by intracerebroventricular infusion of ouabain might be more appropriate. In this study, we confirmed that renal nerves played an important role in the development of ouabain-induced hypertension. However, we could not elucidate the therapeutic effect of RDN on established hypertensive rat models induced by ouabain. Therefore, the antihypertensive and target organ protective effects of RDN on established ouabain-induced hypertensive rats need to be further confirmed. In this study, the mechanism involved in the therapeutic efficacy of RDN on this hypertensive model is unclear which needs to be further explored. Last but not least, it is important to compare the RDN efficacy in this ouabain-induced hypertensive rat model with the ouabain-independent hypertensive rat model to see if the RND efficacy is ouabain-dependent or not. Clinical studies are also needed to verify the antihypertensive efficacy of RDN in hypertensive individuals with or without elevated plasma ouabain levels to help decision-making of rational clinical RDN use.

Overall, RDN inhibited the pressor effect and the myocardial as well as vascular remodeling induced by ouabain independent of the RAAS pathway. Our results favor the possibility that RDN may exhibit a better antihypertensive effect on those hypertensive patients with high plasma ouabain levels. Furthermore, since the blood-pressure-lowering effect of RDN on salt-sensitive hypertensives is controversial [51], our study indicated that RDN might exert a stable antihypertensive effect on salt-sensitive hypertensives with elevated ouabain levels. Our study might thus highlight the importance of exploring the clinical RDN use in patients with high plasma ouabain levels.

## 5. Conclusions

Our study reveals that renal nerves play an important role in the development of ouabain-induced hypertension. RDN inhibited the pressor effect and the myocardial remodeling induced by ouabain potentially via inhibiting catecholamine release and vascular smooth muscle cell proliferation independent of the RAAS pathway. Clinical studies are warranted to know if RDN may exhibit better antihypertensive effects on hypertensive patients with high plasma ouabain levels compared to those with normal plasma ouabain levels [52].

## Figures and Tables

**Table 1:** Echocardiographic parameters of rats after ouabain administration for eight weeks.

Parameters	Control group *n* = 10	RDN group *n* = 10	Ouabain group *n* = 10	Ouabain + RDN group *n* = 10
IVSd (mm)	2.39 ± 0.03	2.22 ± 0.11	2.37 ± 0.12	2.30 ± 0.08
IVSs (mm)	3.61 ± 0.12	3.61 ± 0.12	3.70 ± 0.14	3.73 ± 0.15
LVEF (%)	80.82 ± 3.07	82.12 ± 1.57	82.14 ± 2.30	81.97 ± 1.62
LVFS (%)	51.42 ± 3.28	52.81 ± 1.70	52.95 ± 2.60	52.25 ± 1.84
LVEDd (mm)	5.85 ± 0.16	6.01 ± 0.29	6.46 ± 0.24	6.36 ± 0.18
LVEDs (mm)	2.79 ± 0.25	2.76 ± 0.21	3.25 ± 0.25	3.11 ± 0.21
LVPWd (mm)	2.38 ± 0.12	2.25 ± 0.10	2.43 ± 0.07	2.40 ± 0.14
LVPWs (mm)	3.53 ± 0.14	3.47 ± 0.13	3.56 ± 0.08	3.62 ± 0.16
LV mass (mg)	844.74 ± 37.92	837.44 ± 48.51	970.60 ± 23.21^*∗*^	913.42 ± 29.86

One-way ANOVA, *n* = 10/group. Data presented as mean ± SEM. ^*∗*^*P* < 0.05 versus the control group. IVSs, interventricular septal thickness at end-systole; IVSd, interventricular septal thickness at end-diastole; LVPWs, left ventricular posterior wall systolic thickness; LVPWd, left ventricular posterior wall diastolic thickness; LVESd, left ventricular end-systolic diameter; LVEDd, left ventricular end-diastolic diameter; LVEF, left ventricular ejection fraction; LVFS, left ventricular fractional shortening; LV mass, left ventricular mass.

## Data Availability

The datasets used and/or analyzed during the current study are available from the corresponding author upon reasonable request.

## Abbreviations

RDN: Renal denervation
SNS: Sympathetic nervous system
RAAS: Renin-angiotensin-aldosterone system
Ang II: Angiotensin II
SBP: Systolic blood pressure
DBP: Diastolic blood pressure
SHR: Spontaneous hypertensive rat
VPR: Volume pressure recording
LV: Left ventricle
LVPWd: Posterior wall diastolic thickness of left ventricle
LVPWs: Posterior wall systolic thickness of left ventricle
IVSd: Interventricular septal thickness at end-diastole
IVSs: Interventricular septal thickness at end-systole
LVEDd: Left ventricular end-diastolic diameter
LVESd: Left ventricular end-systolic diameter
LVFS: Left ventricular fractional shortening
LVEF: Left ventricular ejection fraction
HE: Hematoxylin-eosin
TH: Tyrosine hydroxylase
RIA: Radioimmunoassay
LC-MS/MS: Liquid chromatography-tandem mass spectrometry
PCNA: Proliferating cell nuclear antigen
NIH: National Institutes of Health.
